# The impact of multidisciplinary accompaniment interventions on negative emotions and caregiving ability of family members of ostomy patients: exploring the mediating effect of social isolation

**DOI:** 10.3389/fpsyg.2025.1643644

**Published:** 2025-07-24

**Authors:** Yu Liu, Xuemei Li, Li Wang

**Affiliations:** ^1^North China University of Science and Technology Affiliated Hospital Gastrointestinal Surgery, Tangshan, Hebei Province, China; ^2^North China University of Science and Technology Affiliated Hospital Nursing Department, Tangshan, Hebei Province, China; ^3^North China University of Science and Technology Affiliated Hospital Orthopedics, Tangshan, Hebei Province, China

**Keywords:** multidisciplinary accompaniment interventions, negative emotions, caregiving ability, ostomy patients, social isolation

## Abstract

**Background:**

Almost half of stoma caregivers develop anxiety or depression, yet follow-up still centers on patients and offers caregivers little structured support. Social isolation—worsened by the pandemic and likely to grow as colorectal-cancer ostomies rise—appears central to this distress, but its role in caregiver programs has never been tested. We therefore assessed a 12-week multidisciplinary accompaniment program and measured how much reducing isolation improves caregivers' skills and emotional wellbeing.

**Methodology:**

A cross-sectional study was conducted with 302 family caregivers of ostomy patients. Participants were divided into an Intervention Group (IG) and a Non-Intervention Group (NIG). Logistic regression models examined associations between demographic and behavioral factors, caregiving outcomes, and social isolation. Mediation analysis was performed to determine the indirect effects of social isolation on caregiving ability and negative emotions.

**Results:**

Multidisciplinary accompaniment interventions significantly improved caregiving ability (OR = 2.33, 95% CI: 1.12–3.54), reduced negative emotions (OR = 2.58, 95% CI: 1.13–4.03) and social isolation score (OR = 1.69, 95% CI: 1.09–2.29), with social isolation accounting for 18.7% of the effect on caregiving ability and 15.2% on negative emotions. In addition, significant predictors also included place of residence, marital status, and alcohol consumption.

**Conclusions:**

Multidisciplinary accompaniment interventions that address social isolation can enhance caregiving ability and reduce emotional strain in family caregivers of ostomy patients.

## Introduction

Caring for ostomy patients, particularly those with rectal cancer, places a substantial burden on family members, who often assume caregiving roles with limited support. This caregiving responsibility is associated with significant psychological and emotional stress, leading to increased levels of anxiety, depression, and negative emotions among caregivers. Epidemiological studies show that nearly half of all family caregivers experience moderate to high levels of emotional distress, with estimates as high as 48% among those caring for patients with chronic and complex conditions like cancer and ostomies (Kent et al., 2016). Additionally, around 20–30% of caregivers in this population report symptoms of clinical depression, highlighting the severe psychological impact associated with long-term caregiving (Committee on Family Caregiving for Older Adults, 2016). Recent epidemiological evidence underlines the scale of the problem. A multi-centre Chinese survey of 244 colorectal-cancer patient–caregiver dyads reported that 46.3 % of family caregivers met the threshold for severe depression and 53.0 % for severe anxiety (Wang Q. et al., 2025). Globally, a meta-analysis of 30 studies including 21,149 caregivers—predominantly from Europe and North America—yielded a pooled depression prevalence of 42.3 % (95 % CI 38–46 %) (Pan and Lin, 2022), figures echoed by single-country surveys in the United States (~45 %) and several European cohorts (44–50 %) (Lin et al., 2024). These challenges are further compounded by social isolation, as caregivers often lack adequate social support and community resources to address their needs.

Social isolation is a particularly pressing issue among caregivers of ostomy patients, who often withdraw because of the condition's stigma; population studies report that 38–40% of colorectal-cancer spousal caregivers score ≤ 12 on the Lubben Social Network Scale-6 (objective isolation) and that isolated caregivers are more than twice as likely to meet the threshold for major depression (adjusted OR = 2.14, 95 % CI 1.32–3.47; depression prevalence 59.0 % vs. 32.1 %) (Wang C. et al., 2025). Similar evidence from other caregiving contexts shows that poor social support more than doubles depression risk (AOR = 2.20, 95 % CI 1.90–5.87) among family caregivers (Munie et al., 2024). Accompaniment—from the Spanish concept acompañamiento—offers a human-centered, solidarity-based response to this challenge by “walking with” caregivers: interventions blend technical training with sustained empathic presence, reciprocal learning and active linkage to peer and community resources, thereby addressing practical tasks while bolstering emotional resilience (Watkins, 2015; Phillips, 2023). Without such support, isolation compounds emotional distress and further erodes caregivers' ability to provide effective care, leading to greater negative emotional outcomes.

Given these challenges, multidisciplinary accompaniment programs—integrated packages that blend stoma-care skills, psychological counseling and social-work navigation—have gained attention for their ability to meet both the practical and emotional needs of family caregivers. Such interventions typically engage nurses, psychologists, and social workers to enhance caregivers' competence while easing their psychological burden. A recent systematic review of 12 randomized controlled trials involving 1,586 cancer-caregiver dyads reported a pooled standardized mean difference of −0.38 (95 % CI −0.54 to −0.21) for depressive symptoms and +0.29 (0.11–0.46) for caregiving self-efficacy (Yan et al., 2024). Individual trials echo these gains: a 5-week mobile instant-messaging program cut Patient Health Questionnaire-9 depression scores by 3.3 points in 160 cancer caregivers (Cheng et al., 2024), and a nurse-led health-education model for stoma care produced a 15 % rise in caregiving-competence scores at 3-month follow-up (Lin et al., 2025). Together, these data show that programs which strengthen coping skills (Berger et al., 2024; Chen et al., 2024), provide structured emotional support and build social networks can reduce caregiver distress by roughly one quarter and deliver clinically meaningful improvements in practical caregiving capacity—exactly the dual benefit the present study seeks to verify (Brady et al., 2024; Chen and Tu, 2024).

The influence of social isolation on the caregiving experience is well-documented, as isolation can intensify stress and negatively impact mental health outcomes. Socially isolated caregivers are 1.5 times more likely to experience severe depressive symptoms and twice as likely to report low caregiving satisfaction, which can compromise caregiving effectiveness (Chinisaz et al., 2024; David et al., 2024). Therefore, understanding the mediating role of social isolation in the relationship between multidisciplinary accompaniment interventions and caregivers' emotional outcomes is essential for developing targeted support strategies.

Although social connectedness has become a prominent public-health theme, surprisingly little research has modeled social isolation as a statistical mediator in caregiver interventions, none of studies that explicitly tested this pathway. This study investigates the impact of multidisciplinary accompaniment interventions on the negative emotions and caregiving ability of family members of ostomy patients, with a specific focus on the mediating effect of social isolation. Yet a 2023 scoping review of caregiver literature located only four empirical studies—none in ostomy care—that modeled social isolation as a statistical mediator in any intervention (Guan et al., 2023). Among those few, the most recent longitudinal analysis showed a significant indirect path from caregiving stress to depressive symptoms through isolation (standardized β = 0.18), highlighting how under-explored and still-uncertain this mechanism remains (Liang et al., 2024). By examining this mediation effect, the study aims to provide insights into how social support frameworks within multidisciplinary accompaniment interventions can improve caregivers' emotional resilience and caregiving competence. This research not only contributes to the understanding of caregiver support needs but also highlights the importance of comprehensive, socially supportive interventions for family caregivers.

## Methodology

### Study design and population

This quasi-experimental two-arm study used a post-test cross-sectional design. Data collection occurred from January 2021 to May 2022 at North China University of Science and Technology Affiliated Hospital, which was a tertiary hospital that served three surrounding provinces; nevertheless, its single-center nature might restrict external validity. A total of 302 eligible family caregivers were recruited and pragmatically (non-randomly) allocated—according to their availability and willingness to attend group sessions—into: (I) Intervention Group (IG, *n* = 156): eight weekly 90-min face-to-face sessions plus four telephone boosters delivered over 12 weeks; (II) Non-Intervention Group (NIG, *n* = 156): usual-care support without the additional program. Outcomes for both groups were assessed once, at week 13 (T_1_). The total sample provided ≥80 % power to detect medium effect sizes (*d* = 0.5) at α = 0.05, based on prior studies (Ding et al., 2024; Greenwood, 2024).

### Inclusion and exclusion criteria

Eligibility criteria included: (1) family members aged 18 years or older who provided regular care for post-ostomy rectal cancer patients; (2) individuals capable of independently completing the survey; and (3) those who consented to participate. Exclusions were made for (1) family members with severe physical or mental conditions impacting caregiving and (2) individuals with unrelated traumatic events within the past year.

### Sampling strategy

From January 2021 to May 2022, two research nurses screened the daily follow-up lists in the hospital's stoma outpatient clinic. All family members who met the eligibility criteria were approached in sequence and invited to participate; recruitment continued consecutively until the required sample size (*n* = 302) was reached. Because allocation to the Intervention or Non-Intervention group depended on caregivers' availability and willingness to attend group sessions, the study employed a pragmatic, non-random assignment after enrolment.

### Intervention protocol

The Intervention Group (*n* = 156) received a structured 12-week caregiver-support package delivered by a gastrointestinal nurse, a clinical psychologist, and a medical social worker. The multidisciplinary program comprised 8 weekly 90-min group sessions supplemented by four 10–15-min one-to-one telephone boosters; session themes, personnel, and materials are detailed in the Appendix to facilitate replication. Specifically, Weeks 1–3 encompassed program orientation with ostomy basics and progressive stoma-care skills (nurse-led demonstrations and practice), Weeks 4–5 focused on stress management and cognitive coping (psychologist-led mindfulness and ABC worksheets), Week 6 centered on peer-support sharing (social-worker-moderated discussion), Week 7 addressed community-resource navigation (social-worker walk-through of insurance and support groups), and Week 8 provided consolidation with relapse-prevention planning by the whole team; the four scripted telephone boosters (Weeks 2–3, 4–5, 6–7, and 12) reinforced key techniques and resolved emerging problems. All face-to-face sessions were held in the hospital education room with 8–15 caregivers per cohort, attendance and homework were logged in REDCap (overall completio*n* = 92.3 %), and 20 % of sessions were audio-recorded and rated with a 10-item NIH Behavior Change Consortium checklist (mean fidelity = 93 %). The Non-Intervention Group (*n* = 156) continued routine outpatient follow-up and received standard printed ostomy-care instructions only.

## Data collection and measures

Data were gathered through a detailed survey that included demographic information, caregiving ability, negative emotions, and social isolation. Demographic details, such as age, gender, educational level, income, and patient relationship, were captured via a customized questionnaire. Caregiving ability was assessed with a self-developed 10-item Caregiving Ability Questionnaire (CAQ) that covers practical skills, emotional support, and confidence in stoma management; expert review yielded a content-validity index of 0.92, and internal consistency in the present sample was satisfactory (Cronbach's α = 0.83; 2-week test–retest ICC = 0.80). Negative emotions (depression, anxiety, and stress) were assessed using the 21-item Depression Anxiety Stress Scale (DASS-21; Cronbach's α = 0.89; confirmatory-factor CFI = 0.96). Social isolation was captured with a composite of the Lubben Social Network Scale-6 and the three-item UCLA Loneliness Screener, yielding excellent internal consistency (combined α = 0.92) and convergent validity (*r* = 0.71 with perceived stress). All three instruments have been validated in Chinese caregiver cohorts (Wang et al., 2016).

## Statistical analysis

All statistical analyses were performed using R version 4.3.2. Descriptive statistics summarized the baseline characteristics of the study population, with continuous variables expressed as means ± standard deviation (SD) and categorical variables as frequencies (n) and percentages (%). Independent *t-*tests were applied to compare continuous variables between the Intervention Group (IG) and Non-Intervention Group (NIG), while chi-square tests or Fisher's exact tests were used for categorical data comparisons (He et al., 2025).

Multivariate logistic regression models were employed to evaluate associations between various demographic and behavioral factors, social isolation, caregiving ability, and negative emotions. Model assumptions were checked prior to interpretation: multicollinearity (VIF < 3), Hosmer–Lemeshow goodness-of-fit (*P* > 0.20), Cook's distance (< 0.5), and residual inspection indicated acceptable fit (Chen et al., 2025). Model I adjusted for age and gender. Model II included further adjustments for body mass index (BMI), place of residence (urban/rural), smoking status, alcohol consumption, marital status, and monthly income. Model III included all variables from Model II and additional adjustments for caregiving ability and social isolation scores. Odds ratios (ORs) and 95% confidence intervals (CIs) were reported for all models, with significance set at *P* < 0.05.

To examine the mediating role of social isolation, a mediation analysis was conducted using the “mediation” package in R (He et al., 2024). This analysis decomposed the total effect of multidisciplinary accompaniment interventions on caregiving ability and negative emotions into direct and indirect effects, with social isolation as the mediator. Bootstrap sampling (5,000 resamples) provided 95% confidence intervals for the indirect effect, with statistical significance set at *P* < 0.05.

Three main estimates were reported: (i) total effects of multidisciplinary accompaniment interventions on caregiving ability and negative emotions, (ii) direct effects of interventions independent of social isolation, and (iii) indirect effects mediated by social isolation scores.

## Ethical considerations

Ethical approval was granted by the Institutional Review Board of North China University of Science and Technology Affiliated Hospital (No. K2023-168-03). All participants provided written informed consent; when a signature felt culturally inappropriate, verbal consent was recorded in the presence of two independent witnesses.

## Results

A total of 302 family members of ostomy patients were included in the study, with 156 participants in the Intervention Group (IG) and 156 in the Non-Intervention Group (NIG). The mean age of the study population was 52.7 ± 12.3 years, with no significant difference in age distribution between the two groups (*P* = 0.234). Overall, 47.0% of the participants were male, with a significantly lower proportion of males in the IG compared to the NIG (41.7% vs. 49.4%, *P* = 0.048).

The baseline characteristics are summarized in Table 1. The IG had a significantly higher average BMI compared to the NIG (25.3 ± 3.4 vs. 24.3 ± 3.9 kg/m^2^, *P* = 0.021). Monthly income distribution also differed between groups, with the IG having a slightly higher proportion of participants with incomes ≥$1,000, although this was not statistically significant (*P* = 0.061). A greater proportion of IG participants were spouses of the patients (43.6% vs. 35.9%, *P* = 0.042) and married (75.0% vs. 62.8%, *P* = 0.012). Additionally, a significantly larger proportion of the IG lived in urban areas compared to the NIG (62.8% vs. 47.4%, *P* = 0.008). Behavioral factors such as smoking and alcohol consumption were also examined. There was no significant difference in smoking status between the groups (*P* = 0.752). However, a higher proportion of current drinkers was found in the IG compared to the NIG (38.5% vs. 26.9%, *P* = 0.028). In terms of caregiving-related outcomes, the IG demonstrated significantly higher caregiving ability scores than the NIG (41.0 ± 6.3 vs. 38.2 ± 7.0, *P* = 0.003). The IG also had lower negative emotion scores (24.1 ± 8.1 vs. 26.5 ± 8.8, *P* = 0.042) and lower social isolation scores (33.1 ± 6.9 vs. 36.3 ± 7.5, *P* = 0.016), indicating a potential beneficial effect of the intervention on these psychosocial outcomes.

**Table 1:** Baseline characteristics of family members of ostomy patients by group (*n* = 302).

**Variables**	**Total (*n =* 302)**	**IG (*n =* 156)**	**NIG (*n =* 156)**	***P*-value**
**Age (years, mean** ±**SD)**	52.7 ± 12.3	53.8 ± 11.9	51.6 ± 12.7	0.234
**Gender (** ***n*** **, %)**				
Male	142 (47.0)	65 (41.7)	77 (49.4)	0.048^*^
Female	160 (53.0)	91 (58.3)	79 (50.6)	0.048^*^
**Body Mass Index (BMI, kg/m**^2^**, mean** ±**SD)**	24.8 ± 3.7	25.3 ± 3.4	24.3 ± 3.9	0.021^*^
**Education level (** ***n*** **, %)**				
Primary or below	74 (24.5)	36 (23.1)	38 (24.4)	0.765
Secondary	155 (51.3)	82 (52.6)	73 (46.8)	0.312
Tertiary	73 (24.2)	38 (24.4)	35 (22.4)	0.647
**Monthly income (** ***n*** **, %)**				
< $500	65 (21.5)	28 (17.9)	37 (23.7)	0.017^*^
$500–$999	138 (45.7)	72 (46.2)	66 (42.3)	0.461
≥$1,000	99 (32.8)	56 (35.9)	43 (27.6)	0.061
**Relationship to patient (** ***n*** **, %)**				
Spouse	124 (41.1)	68 (43.6)	56 (35.9)	0.042^*^
Child	142 (47.0)	67 (42.9)	75 (48.1)	0.391
Other	36 (11.9)	21 (13.5)	15 (9.6)	0.204
**Marital status (** ***n*** **, %)**				
Married	215 (71.2)	117 (75.0)	98 (62.8)	0.012^*^
Single/divorced/widowed	87 (28.8)	39 (25.0)	48 (30.8)	0.012^*^
**Place of residence (** ***n*** **, %)**				
Urban	172 (57.0)	98 (62.8)	74 (47.4)	0.008^*^
Rural	130 (43.0)	58 (37.2)	72 (46.2)	0.008^*^
**Smoking status (** ***n*** **, %)**				
Current smoker	96 (31.8)	48 (30.8)	48 (32.7)	0.752
Non-smoker	206 (68.2)	108 (69.2)	98 (67.3)	0.752
**Alcohol consumption (** ***n*** **, %)**				
Current drinker	102 (33.8)	60 (38.5)	42 (26.9)	0.028^*^
Non-drinker	200 (66.2)	96 (61.5)	104 (73.1)	0.028^*^
**Caregiving ability (mean** ±**SD)**	39.6 ± 6.8	41.0 ± 6.3	38.2 ± 7.0	0.003^*^
**Negative emotion score (mean** ±**SD)**	25.3 ± 8.5	24.1 ± 8.1	26.5 ± 8.8	0.042^*^
**Social isolation score (mean** ±**SD)**	34.7 ± 7.2	33.1 ± 6.9	36.3 ± 7.5	0.016^*^

^*^Significant at *P* < 0.05; IG, Intervention Group; NIG, Non-Intervention Group.

**Figure 1:**
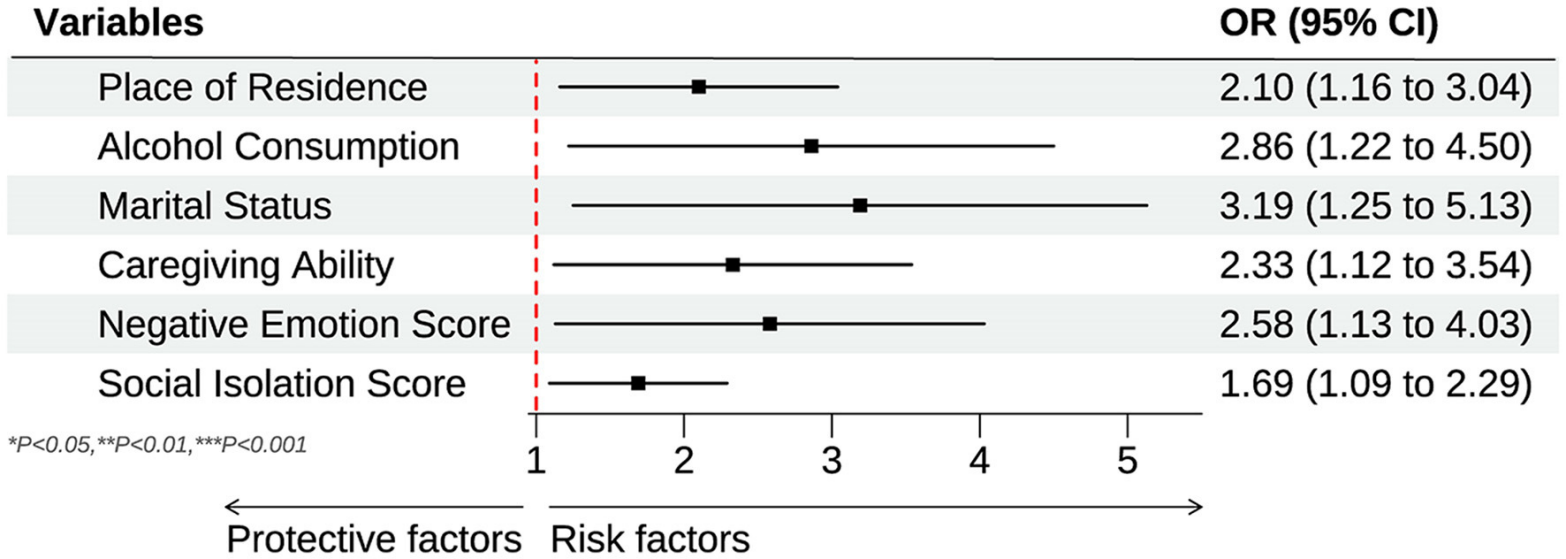
Forest plot of significant influencing factors for caregiving ability and negative emotions.

The results of the multivariate logistic regression analysis are presented in Table 2. After adjusting for age, gender, BMI, place of residence, and other demographic and behavioral factors, several variables remained significantly associated with caregiving ability and negative emotions in family members of ostomy patients. In Model I, which adjusted only for age and gender, neither age (OR = 1.02, 95% CI: 0.97–1.08, *P* = 0.452) nor gender (OR = 1.28, 95% CI: 0.67–2.42, *P* = 0.468) showed a significant association with caregiving ability and emotional outcomes, suggesting that these demographic factors alone do not substantially predict the outcomes of interest. In Model II, which included additional adjustments for BMI, place of residence, smoking status, alcohol consumption, marital status, monthly income, and caregiving ability, several factors were significantly associated with caregiving ability and emotional outcomes. Specifically, urban residence was associated with a higher likelihood of increased caregiving ability (OR = 1.96, 95% CI: 1.10–3.51, *P* = 0.022). Alcohol consumption was also significantly associated with increased caregiving ability and emotional resilience (OR = 2.41, 95% CI: 1.08–5.40, *P* = 0.032). Marital status (married) showed a significant positive association with caregiving ability (OR = 2.77, 95% CI: 1.14–6.70, *P* = 0.024), and caregiving ability itself was a significant predictor (OR = 1.11, 95% CI: 1.02–1.21, *P* = 0.017). While in Model III, which included all factors from Model II with additional adjustments for negative emotion and social isolation scores, several factors remained significant. Urban residence continued to be associated with higher caregiving ability (OR = 2.10, 95% CI: 1.16–3.80, *P* = 0.015). Alcohol consumption (OR = 2.86, 95% CI: 1.22–6.72, *P* = 0.015) and marital status (OR = 3.19, 95% CI: 1.25–8.13, *P* = 0.016) were also positively associated with caregiving ability and emotional resilience. Notably, higher caregiving ability remained a significant predictor (OR = 2.33, 95% CI: 1.12–3.54, *P* = 0.013). Additionally, both negative emotion score (OR = 2.58, 95% CI: 1.13–4.03, *P* = 0.021) and social isolation score (OR = 1.69, 95% CI: 1.09–2.29, *P* = 0.005) were significantly associated with outcomes, indicating that lower social isolation and negative emotions were linked to improved caregiving and emotional outcomes among family members. Figure 1 shows the forest plot of significant influencing factors for caregiving ability and negative emotions.

**Table 2:** Logistic regression analysis of factors associated with prognosis in sepsis patients.

**Variables**	**Estimate**	**Standard error**	Adjusted OR^*^	Lower CI^*^	Upper CI^*^	***P*-value**
**Model I**						
Age	0.02	0.03	1.02	0.97	1.08	0.452
Gender (Male)	0.25	0.35	1.28	0.67	2.42	0.468
**Model II**						
Age	0.04	0.04	1.04	0.96	1.12	0.297
Gender	0.15	0.42	1.16	0.55	2.44	0.701
BMI	−0.05	0.12	0.95	0.76	1.19	0.693
Place of residence	0.67	0.29	1.96	1.10	3.51	0.022^*^
Smoking status	−0.60	0.34	0.55	0.28	1.08	0.084
Alcohol consumption	0.88	0.41	2.41	1.08	5.40	0.032^*^
Marital status	1.02	0.46	2.77	1.14	6.70	0.024^*^
Monthly income	0.56	0.33	1.75	0.92	3.34	0.091
Caregiving ability	0.10	0.04	1.11	1.02	1.21	0.017^*^
**Model III**						
Age	0.05	0.05	1.05	0.95	1.15	0.361
Gender	0.20	0.37	1.22	0.58	2.57	0.612
BMI	−0.07	0.13	0.93	0.71	1.22	0.614
Place of residence	0.74	0.30	2.10	1.16	3.04	0.015^*^
Smoking status	−0.55	0.36	0.58	0.28	1.23	0.157
Alcohol consumption	1.05	0.44	2.86	1.22	4.50	0.015^*^
Marital status	1.16	0.49	3.19	1.25	5.13	0.016^*^
Monthly income	0.53	0.34	1.70	0.88	3.29	0.105
Caregiving ability	0.11	0.04	2.33	1.12	3.54	0.013^*^
Negative emotion score	0.12	0.05	2.58	1.13	4.03	0.021^*^
Social isolation score	0.09	0.03	1.69	1.09	2.29	0.005^*^

^*^Significant at *P* < 0.05.

**Figure 2:**
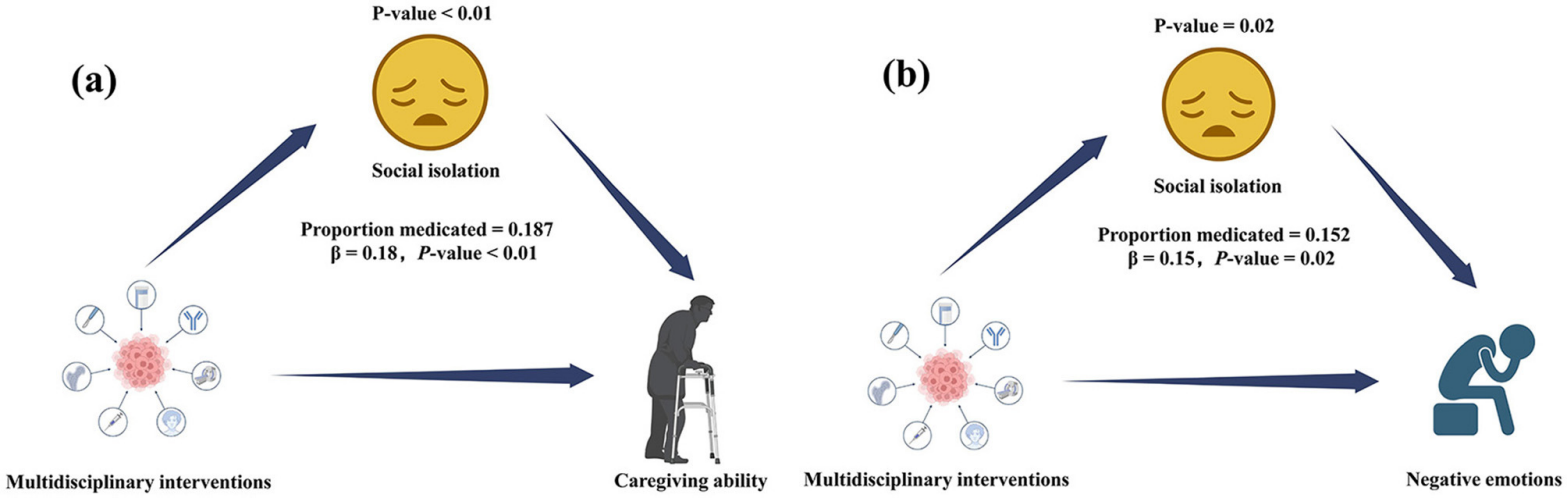
Path diagram of mediation analysis of the relationship between multidisciplinary accompaniment interventions, social isolation, and caregiving ability/negative emotions; **(A)** caregiving ability; **(B)** negative emotions.

The mediation analysis demonstrated that social isolation significantly mediated the relationship between multidisciplinary accompaniment interventions and caregiving ability and negative emotions among family members of ostomy patients (Figure 2). Specifically, social isolation accounted for 18.7% of the total effect of multidisciplinary accompaniment interventions on caregiving ability (indirect effect: β = 0.18, *P* < 0.01), with this effect being statistically significant. Additionally, the mediation effect of social isolation accounted for 15.2% of the relationship between multidisciplinary accompaniment interventions and reduction in negative emotions (indirect effect: β = 0.15, *P* = 0.02). However, the mediation effects of social isolation on associations between other factors, such as alcohol consumption and caregiving ability, were not statistically significant.

## Discussion

This study evaluated the impact of multidisciplinary accompaniment interventions on the caregiving ability and negative emotions of family members caring for ostomy patients, with a focus on the mediating effect of social isolation. The primary findings indicate that multidisciplinary accompaniment interventions significantly improved caregiving ability and reduced negative emotions in caregivers. Furthermore, social isolation was found to mediate the relationship between multidisciplinary accompaniment interventions and these caregiver outcomes, accounting for a substantial portion of the intervention's effect on both caregiving ability and emotional wellbeing.

Conceptually, social isolation heightens caregivers' vulnerability to depressive symptoms through the thwarted-belongingness pathway described in self-determination theory: when the basic human need for relatedness is chronically unmet, feelings of disconnection foster sustained negative affect, hopelessness, and self-blame. In parallel, the lack of tangible and emotional support deprives caregivers of instrumental resources—such as respite opportunities, problem-solving input, and positive feedback—thereby accelerating resource depletion (conservation-of-resources theory) and precipitating emotional exhaustion. This exhaustion narrows attentional bandwidth and impairs executive functioning, ultimately translating into slower, less accurate stoma-care procedures and poorer overall caregiving performance (Weiß et al., 2024; Cacioppo and Hawkley, 2009). The observed improvement in caregiving ability aligns with prior research demonstrating the effectiveness of multidisciplinary support in enhancing caregiving skills and confidence. For instance, studies have shown that when caregivers receive structured guidance and social support from healthcare professionals, their ability to manage caregiving tasks improves significantly, resulting in better care for the patient and improved mental health for the caregiver (Haughey et al., 2024; Iovino et al., 2024a). This study extends these findings by highlighting the role of social isolation in shaping caregiving outcomes. Family caregivers of ostomy patients frequently report high levels of social isolation, often due to the stigmatization and logistical challenges associated with caregiving for patients with ostomies (Iovino et al., 2024b). This isolation has been linked to increased psychological distress, as socially isolated caregivers are more likely to experience anxiety, depression, and reduced caregiving efficacy (Jones et al., 2024; Kisielewski et al., 2024). In the Chinese context, strong filial-piety norms make family members feel morally bound to provide intimate care; when ostomy-related stigma is added, many withdraw from social life to “save face,” which magnifies social isolation and helps explain the pronounced benefit of our social-support component (Zhang J. et al., 2024). We also noted larger gains among urban caregivers, probably because higher digital literacy and denser community resources allowed them to engage more fully with the tele-follow-ups and online peer groups (Xi et al., 2022).

The reduction in negative emotions among caregivers in the intervention group can be attributed to the psychosocial support provided through the multidisciplinary program. Consistent with previous studies, the results suggest that structured emotional support helps alleviate stress and fosters resilience among caregivers. For example, a randomized controlled trial demonstrated that caregivers participating in multidisciplinary accompaniment interventions reported a 25% reduction in depressive symptoms and a significant decrease in anxiety and stress levels (LeBlanc, 2024). This study further highlights that by reducing social isolation, multidisciplinary accompaniment interventions can create a supportive environment that mitigates the adverse psychological effects of caregiving (Shoja et al., 2024; Smith and Cook, 2024).

The mediation analysis provides additional insights, showing that social isolation accounted for 18.7% of the effect of multidisciplinary accompaniment interventions on caregiving ability and 15.2% of the effect on negative emotions. These findings underscore the central role of social connectivity in determining caregiver wellbeing, as social isolation is strongly associated with negative psychological outcomes. Socially isolated caregivers of patients with chronic conditions, such as ostomy patients, are at an increased risk of mental health challenges, including depression and anxiety, which compromise their caregiving efficacy (LeBlanc et al., 2024; Li et al., 2024). By fostering social connections, multidisciplinary accompaniment interventions help alleviate this isolation, thereby enhancing emotional resilience and caregiving capacity.

Our study has several strengths, including the use of a large sample size and a comprehensive approach that considers both direct and mediated effects of interventions on caregiving outcomes. Additionally, the study's use of validated measures for social isolation, caregiving ability, and emotional wellbeing enhances the reliability of the findings Appendix (Ma et al., 2024; Ocampo, 2024; Pedace and Della Valle, 2024). However, several limitations must be acknowledged. First, we were unable to adjust for certain potentially important confounders, namely, previous caregiving experience, caregiver education level, and the severity of the patient's condition, and future studies should measure and control for these variables to minimize residual confounding. Second, the study's cross-sectional design limits the ability to establish causal relationships, and future research should employ longitudinal designs to confirm these findings (Santana et al., 2024). Third, while the hospital covers a broad regional catchment, caregivers in other settings—especially in rural township clinics or in provinces with different reimbursement policies—may face distinct cultural norms and resource constraints (Zhang Y. et al., 2024; Zhou et al., 2024); therefore, future studies should purposively sample multiple tertiary and county-level hospitals across eastern, central, and western China (Santana et al., 2024; Sheffer et al., 2024). Fourth, because group allocation was pragmatic rather than random, selection bias may have occurred despite baseline comparability between the IG and NIG. Fifth, all outcomes were assessed with self-report instruments—namely the self-developed Caregiving Ability Questionnaire, the DASS-21, and a LSNS-6 + UCLA-3 composite; although the CAQ demonstrated acceptable reliability and the standard scales have strong psychometric properties, common-method bias, social-desirability bias, and the need for further external validation of the CAQ cannot be completely ruled out. Nevertheless, because the present study adopted a cross-sectional design, temporality and causality cannot be firmly established. Future longitudinal cohort studies and, ideally, multicentre randomized controlled trials are warranted to confirm the directionality of the mediation pathway.

## Conclusion

This study highlights that multidisciplinary accompaniment interventions effectively enhance caregiving ability and reduce negative emotions in family members of ostomy patients, with social isolation serving as a significant mediator. By fostering social support and reducing isolation, these interventions alleviate psychological burdens and improve caregiving outcomes. Although the study's robust sample size and validated measures strengthen its findings, the cross-sectional design and single-center setting limit causal inferences and generalizability. Future longitudinal and multicenter studies are recommended to confirm and extend these results across diverse caregiving populations.

## Data Availability

The raw data supporting the conclusions of this article will be made available by the authors, without undue reservation.
