# Supplementary material for: The impact of multidisciplinary accompaniment interventions on negative emotions and caregiving ability of family members of ostomy patients: exploring the mediating effect of social isolation
Source: Front Psychol. 2025 Jul 24;16:1643644. doi: 10.3389/fpsyg.2025.1643644 (PMC12328384; doi:10.3389/fpsyg.2025.1643644)
Supplement: Supplementary file 1 [file Supplementary_file_1.docx]

| **Appendix.** Multidisciplinary Caregiver-Support Programme Syllabus (Intervention Group) | | | | | | | |
| --- | --- | --- | --- | --- | --- | --- | --- |
| **Week / Session** | **Core Theme & Objectives** | **Key Activities / Content Outline** | **Facilitator(s)*** | **Delivery Mode** | **Materials / Resources Supplied** | **Home Practice / Assignments** | **Duration** |
| **1** | **Programme orientation & baseline needs assessment**  • Build rapport  • Introduce ostomy basics & programme schedule | • Ice-breaker & caregiver role mapping • Short lecture: anatomy of stoma, pouch system, daily routine • Baseline stress / isolation scales administered | GI Nurse (lead) Social Worker | Face-to-face group (8-12 caregivers) | Illustrated booklet “Living With a Stoma” • Caregiver diary (blank) | Record daily challenges (min. 3 days) | 90 min |
| **2** | **Stoma-care skills I: pouch change & skin hygiene**  • Master aseptic technique  • Reduce leakage incidence | • Live demonstration on mannequin • Hands-on practice with feedback • Troubleshooting Q&A | GI Nurse | Face-to-face | Mannequin, standard ostomy kits, step-by-step video (USB) | Practise pouch change at home & log time taken | 90 min |
| **3** | **Stoma-care skills II: complication prevention & early detection**  • Identify common peristomal problems  • Develop action plan | • Photo gallery of complications (dermatitis, retraction, prolapse) • Small-group scenario discussion • Red-flag checklist distribution | GI Nurse | Face-to-face | Slide set • “Red-Flag” fridge magnet | Tick checklist weekly; call nurse if ≥1 red flag | 90 min |
| **4** | **Stress-management & mindfulness training**  • Lower physiological arousal  • Introduce mindfulness methods | • Psycho-education on stress response • Guided breathing & 5-minute body-scan audio • Pair share: stress triggers | Clinical Psychologist | Face-to-face | MP3 audio file • Mini-poster “STOP & Breathe” | Daily 5-min breathing exercise; note mood (1-10) | 90 min |
| **5** | **Cognitive coping & emotion regulation**  • Challenge unhelpful thoughts  • Build positive self-talk | • ABC (Activating-Belief-Consequence) worksheet • Role-play disputing negative thoughts • Emotion-labelling drill | Clinical Psychologist | Face-to-face | CBT workbook (translated) | Complete one ABC sheet per stressful event | 90 min |
| **6** | **Peer-support & experience sharing**  • Reduce isolation  • Harness collective wisdom | • Round-table storytelling • Problem-solving in triads • Exchange of practical tips | Social Worker (moderator) | Face-to-face | Whiteboard • Contact-card swap templates | Add two peers to personal support list | 90 min |
| **7** | **Community resources & benefit navigation**  • Connect with social services  • Understand financial support | • Presentation: local ostomy association, online forums • Walk-through of insurance / reimbursement forms • On-site membership sign-up | Social Worker | Face-to-face | Resource directory booklet • QR codes for WeChat groups | Join at least one community group; post greeting | 90 min |
| **8** | **Consolidation & personal action plan**  • Review skills learned  • Plan for relapse prevention | • Group quiz game (“Jeopardy”) • Individualised action-plan worksheet • Feedback survey | All facilitators | Face-to-face | Action-plan template • Certificate of completion | Implement plan; schedule follow-up call | 90 min |
| **Telephone Booster 1** | Progress check (Weeks 2–3) | Structured 6-question script; motivational interviewing | Nurse | Phone | — | Update diary, adjust goals | 10-15 min |
| **Telephone Booster 2** | Progress check (Weeks 4–5) | Review stress log; reinforce mindfulness | Psychologist | Phone | — | Continue breathing practice | 10-15 min |
| **Telephone Booster 3** | Progress check (Weeks 6–7) | Review peer-connections; troubleshoot barriers | Social Worker | Phone | — | Contact support buddy once | 10-15 min |
| **Telephone Booster 4** | Final check & transition (Week 12) | Evaluate action plan; plan long-term follow-up | Nurse + Social Worker | Phone | — | Optional referral if new issues | 10-15 min |

*Facilitators received 4 h joint training to ensure content fidelity and consistent delivery style.

**Notes**

1. All face-to-face sessions were held in the hospital’s education room (max 15 participants, U-shaped seating).
2. Caregivers who missed a group session received a 30-min individual make-up via video call.
3. Attendance, homework completion, and any adverse events were logged in an electronic REDCap database.
4. Fidelity was monitored through random audio recording of 20 % of sessions and evaluated against a 10-item checklist (mean adherence = 93 %).
